# Characterization of Experimental Nanoparticulated Dental Adhesive Resins with Long-Term Antibacterial Properties

**DOI:** 10.3390/nano12213732

**Published:** 2022-10-24

**Authors:** Rochelle Denise Hiers, Pedro Huebner, Sharukh Soli Khajotia, Fernando Luis Esteban Florez

**Affiliations:** 1 Division of Dental Biomaterials, Department of Restorative Sciences, College of Dentistry, University of Oklahoma Health Sciences Center, Oklahoma City, OK 73117, USA; 2 Department of Mechanical Engineering, University of Utah, Salt Lake City, UT 84112, USA

**Keywords:** metal nanoparticles, anti-bacterial agents, dental materials, *Streptococcus mutans*

## Abstract

Experimental adhesives with functional nitrogen-doped titanium dioxide nanoparticles (N_TiO_2_) have been shown to display improved properties. However, these materials have not been characterized regarding their degree of conversion (DC), biaxial flexure strength (BFS), surface roughness (SR), elastic modulus (EM), and long-term antibacterial functionalities. Experimental adhesives were synthesized by dispersing N_TiO_2_ (10%, 20%, or 30%, *v*/*v*%) into OptiBond Solo Plus (OPTB, Kerr Corp., USA). Unpolymerized adhesives (volume = 50 μL/drop, *n* = 3/group) were individually placed onto a heated (37 °C) attenuated total reflectance (ATR) monolithic diamond crystal (Golden Gate, Specac). The spectra of composites were obtained with a Fourier-transform infrared (FTIR) spectrometer (Nicolet IS50; 500–4500 cm^−1^; resolution = 4 cm^−1^, 10 internal scans/spectrum) before and after polymerization. Disk-shaped specimens (diameter = 6.0 mm, thickness = 0.5 mm) for BFS (*n* = 12/group), SR and EM (*n* = 3/group), and for antibacterial testing (*n* = 18/group/time-point) were fabricated and photopolymerized (1 min each; 385–515 nm, 1000 mW/cm^2^; VALO). DC values (%) were calculated from pre- and post-polymerization spectra using the two-frequency method and tangent-baseline technique. BFS was assessed using a universal testing machine (Instron 68TM-5, crosshead speed = 1.27 mm/min, 25 °C). SR and EM were investigated using an atomic force microscope (Multimode 8) with aluminum-coated silicon probes (8 nm pyramidal tip, spring constant 40 N/m, Bruker). Antibacterial testing was performed by growing *Streptococcus mutans* biofilms (UA159-*ldh*, 37 °C, microaerophilic) on the surfaces of specimens for 24 h and then measuring the relative luminescence units (RLU) with a Biotek Synergy HT multi-well plate reader. Results demonstrate that experimental materials containing 10%, 20%, and 30% of N_TiO_2_ displayed higher levels of DC, had better mechanical properties, and were able to exert strong and durable antibacterial properties without visible light irradiation and after extended periods of simulated shelf-life and aging in PBS. The reported experimental materials are expected to increase the service lives of polymer-based bonded restorations by decreasing the incidence of secondary caries.

## 1. Introduction

Dental caries continues to pose a major health burden in numerous countries. This biofilm-originated disease [1] is estimated to affect 3.5 billion people [2] and to represent around 4.6% of the total global expenditures in healthcare [3]. Clinical manifestations include the progressive and irreversible dissolution [4,5] of dental hard tissues (e.g., enamel, dentin, and cementum), cavitation, pain, and tooth loss. The treatment of dental caries revolves around the mechanical removal of disorganized and infected tissues using hand-held instruments (e.g., either static or rotary) and the placement of a dental biomaterial (e.g., metal, polymer, or ceramic) to restore the esthetics and masticatory function of affected tissues.

Over the years, composite resins became the first choice of restorative materials amongst patients and clinicians due to its mercury-free compositions [6] and superior properties (e.g., handling and esthetic) [7,8,9]. In fact, composite resin restorations are the most prevalent biomedical intervention in human beings with more than 800 million placed every year [10]. Despite such widespread acceptance and utilization, previous studies indicated that these materials are associated with limited-service lives (between 5–7 years), [11] experience polymerization shrinkage and accumulate more biofilms, when compared to other restorative materials [9,12,13]. When combined, these factors may shift the ecology of the oral cavity from a healthy state into a disease-associated state [14].

*Streptococcus mutans*, a Gram-positive and facultatively anaerobic bacteria, has been widely accepted as a major contributor to the development of dental caries (primary and secondary) due to their (i) ability to adhere and accumulate onto the surfaces of teeth through a disaccharide-dependent mechanism, (ii) ability to deposit an extracellular matrix that protects cells from external aggressors, (iii) ability to metabolize a wide variety of complex carbohydrates into organic acids, and (iv) the ability to thrive in acidic environments [15]. Even though *S. mutans* is not solely responsible for the occurrence of dental caries, these undisputable features have made *S. mutans* an important model organism in oral antibacterial research [16].

Secondary caries develop between dental adhesive resins and the tooth structure, and are considered the primary reason for the failure of polymer-based bonded restorations [17]. According to previous studies, current dental adhesive resins are formulated using a combination of hydrophilic and hydrophobic components [18] that phase-separate when applied onto water-rich tissues [19]. This chemical instability leads to incomplete envelopment of exposed collagen fibrils and the establishment of porous hybrid layers that are prone to failure by biodegradation, hydrolysis, esterases, and biofilms [20]. This significant problem has precipitated the execution of several studies to improve the physical, chemical, and biological properties of current polymer formulations. Ideally, dental adhesive resins should be able to establish interfaces that are hermetically sealed, are dimensionally stable, prevent the formation of cariogenic biofilms, and precipitate highly organized crystalline structures to fill the gaps from the incomplete envelopment of collagen fibrils and polymerization shrinkage [21,22,23].

Despite significant investments by the manufacturing and scientific communities, newly developed materials containing antibacterial agents, quaternary ammonium compounds, [13] or metaloxide nanoparticles (e.g., zinc and titanium) [24,25] failed to sustain long-term antibacterial properties and did not extend the service lives of composite restorations, thereby underscoring the need for the development of novel materials with long-term biological properties. Pérez-Mondragón et al., [26] while investigating the shelf-life stability in terms of the degree of conversion, ultimate tensile strength, and color of dental adhesive resins (commercially available and experimental) at different periods of simulated shelf-life (37 °C; 6, 18, and 24 months), have demonstrated that experimental materials displayed higher shelf-life stability (in terms of the degree of conversion), when compared to commercially available materials. However, experimental materials displayed mechanical and optical properties that were similar (*p* > 0.05) to those of the control group, independently of the time-point considered [26].

Recent advances in the field of material sciences and nanotechnology enabled the synthesis and incorporation of metaloxide nanoparticles (e.g., zinc oxide, silver, and titanium) into dental polymers (denture base, dental adhesives, and composite resins). These nanoparticulated systems have become prevalent in many areas of dental research, including orthodontics, dental materials, bleaching, implants, and prosthodontics [27], because of their intrinsic optical, physical, biological, and chemical properties [28,29]. Recent studies investigated the utility of silver, zinc, copper, titanium, calcium fluoride, and magnesium nanoparticles [30,31] in the prevention of secondary caries because they have been shown to disrupt bacterial metabolism and biofilm formation [32].

Titanium dioxide (TiO_2_, anatase, rutile, or brookite) is known for its relevant physical, chemical, antimicrobial, and biocompatibility properties [33]. Nanoparticles of TiO_2_ (TiO_2_-np) are typically white, have diameters around 25 nm, have high refractive index, are corrosion resistant, display high microhardness values [20,34], and were demonstrated to be effective against numerous microorganisms, including *Candida albicans*, *Staphylococcus aureus*, *Pseudomonas aeruginosa*, *Escherichia coli*, and *Lactobacillus acidophilus* [35]. However, despite these relevant characteristics, TiO_2_-np have large bandgaps (3.2204 eV, for anatase) and require the utilization of UV irradiation to generate different types of reactive oxygen species (ROS) [34]. Even though the photocatalysis of TiO_2_-np is feasible from the electronic standpoint, the UV dose of energy required to promote surface disinfection has been demonstrated to be harmful to eukaryotic cells and tissues, [36] which significantly restricts its widespread utilization in dental applications.

Doping the crystal lattice of TiO_2_-np with metals and non-metals has been previously shown to decrease the bandgap of Titania (2.47 eV) [37] and allow the utilization of visible light irradiation, which is widely used in dentistry, for the generation of ROS. The synthesis, incorporation, and covalent functionalization of visible light-responsive nitrogen-doped titanium dioxide nanoparticles (N_TiO_2_) into a commercially available dental adhesive resin (OptiBond Solo Plus, Kerr Corp., USA; OPTB) has been recently reported by Esteban Florez et al. [34,38,39] Experimental adhesive resins displayed strong antibacterial and biomimetic properties when irradiated with visible light [34] and were less soluble and more biocompatible [20], when compared to commercially available materials, which suggests that nanoparticulated materials may hold the promise to decrease the incidence of secondary caries and to extend the service lives of polymer-based adhesive restorations.

Therefore, the objective of the present study was to characterize the (i) degree of conversion at the time of polymer synthesis and after two years of simulated shelf-life, (ii) biaxial flexure strength, (iii) flexural modulus, (iv) surface roughness, (v) elastic modulus, and (vi) long-term antibacterial properties of experimental dental adhesive resins, containing varying concentrations of N_TiO_2_ (10%, 20%, and 30%, *v*/*v*%).

## 2. Materials and Methods

### 2.1. Synthesis of N_TiO_2_

Synthesis of N_TiO_2_ nanoparticles has been described in detail in previous publications [17,29,35,36] from our group and will be summarized here. Nitrogen-doped TiO_2_ nanoparticles (N_TiO_2_) were synthesized via a 2-step process. The first step involved a solvothermal synthesis of pure TiO_2_. In a typical reaction, a solution comprised of 1.7 g of Ti (IV)-butoxide (Aldrich, St. Louis, MO, USA, 97%), 4.6 g ethanol (Decon Labs, 200 proof), 6.8 g oleylamine (Aldrich, 70%), and 7.1 g oleic acid (Aldrich, St. Louis, MO, USA, 90%) was prepared, then mixed with 20 mL of 4% H_2_O in ethanol (18-MΩ Milli-Q; Decon Labs, King of Prussia, PA, USA). The solution was reacted using a high-pressure reaction vessel (Paar Series 5000 Multiple Reactor System) and continuous stirring at 180 °C for 24 h. Solutions went through a series of washing steps with anhydrous ethanol to remove extraneous surfactants. The synthesized TiO_2_ nanoparticles were stored in ethanol. To produce nitrogen-doped TiO_2_ nanoparticles, aliquots were then reacted with an equal volume of triethylamine (Aldrich, St. Louis, MO, USA, 99.5%), and underwent a second period in a high-pressure reaction vessel, at 140 °C for 12 h. The N_TiO_2_ particles (size distribution= 6–15 nm, anatase) were rinsed 3 times with anhydrous ethanol. The final N_TiO_2_ nanoparticle solution was stored in ethanol, and the concentration of particles was determined gravimetrically and was approximately 35 mg/mL.

### 2.2. Synthesis of Experimental Adhesive Resins and Specimen Fabrication

Experimental dental adhesive resins were synthesized by dispersing (Q700 sonicator, QSonica, LLC, USA) 10%, 20%, or 30% of N_TiO_2_ (*v*/*v* %, suspended in ethanol) into OptiBond Solo Plus (Kerr Corp., OPTB, Composition: Bis-GMA, HEMA, GDMA, GPDM, ethanol, CQ, ODMAB, BHT, filler particles, and coupling factor A174). The rationale for selecting these concentrations of nanoparticles was based on a previous study where strong initial antibacterial properties were demonstrated in both dark and light-irradiated conditions [34]. Disk-shaped specimens (*n* = 18/group; diameter = 6.00 mm, thickness = 0.50 mm) of unaltered OPTB and experimental dental adhesive resins were fabricated using a custom stainless-steel mold. Glass coverslips (No. 2, VWR International, Radnor, PA, USA, LLC) were used to give specimens a smooth surface finish. Specimens were polymerized using blue light irradiation (1000 mW/cm^2^, 1 min) emitted from a broadband LED light-curing unit (VALO, Ultradent Products, Inc., South Jordan, UT, USA). Specimens of both unaltered and experimental adhesive resins were then UV-sterilized (254 nm, 800,000 µJ/cm^2^, UVP Crosslinker, model CL-1000, UVP, Upland, CA, USA).

### 2.3. Degree of Conversion

Adhesives described in Section 2.2. were assessed for degree of conversion at the time of polymer synthesis (NEW) and after two years of simulated shelf-life (OLD; dark conditions, 25 °C) using an attenuated total reflectance (ATR) diamond crystal (KR-5 lens, Golden Gate model GS10542-K; Specac, Inc. Fort Washington, PA, USA) coupled to a Fourier transform infrared spectrometer (500–4000 cm*^−^*^1^; resolution 4 cm*^−^*^1^, 10 internal scans per spectrum; Nicolet IS50, Thermo Fisher Scientific, Waltham, MA, USA). Uncured drops (volume = 50 μL/drop, *n* = 3/group) of each material were individually dispensed onto the ATR crystal (at 37 °C). Spectra of materials in the unpolymerized state were then recorded. Materials were individually photopolymerized using a broad-band LED light curing unit (385*–*515 nm, 1000 mW/cm^2^, 20 sec., VALO, Ultradent Products, Inc., South Jordan, UT, USA) before obtaining the spectra of materials in the polymerized state. Values of DC reported were calculated using the two-frequency method [40] and tangent-baseline technique [41].

### 2.4. Biaxial Flexure Strength

The biaxial flexure strength (BFS) at the time of polymer synthesis was investigated to test the bulk mechanical properties of experimental adhesive resins. This method was selected because it eliminates spurious edge failures that are typically associated with three-point bending testing and results are independent of flaw direction [42]. Specimens (*n* = 12/group) were fabricated, as described in Section 2.2., and were subjected to BFS testing using a universal testing machine (Instron, model 33R4468, cross-head speed = 1.27 mm/min, 25 °C, Norwood, MA, USA) until failure.

### 2.5. Nanoscale Surface and Mechanical Characterization

The concurrent characterization of surface roughness and elastic modulus of specimens (*n* = 3/group) fabricated, as described in Section 2.2., was conducted using an atomic force microscope (AFM, Multimode 8, Bruker Corporation, Billerica, MA, USA) with aluminum-coated silicon probes (RTESPA-300, Bruker, 8 nm pyramidal tip, spring constant, 40 N/m). In brief, following AFM calibration, as directed by the manufacturer, three discrete 20 × 20 µm areas on the surface of each specimen were randomly selected for characterization. The quantitative nanomechanical (QNM) test was configured to scan the surfaces of samples at a rate of 0.2 Hz and 8 µm/s. Peak forces were limited to 80–120 nN, depending on the specimen, so as to achieve the necessary amount of surface deformation for mechanical data modelling and to protect the tip of the probe from excessive wear. Within each area, square matrices of 256 × 256 equally spaced data points were captured and used to (i) characterize the average surface roughness (Ra) measured in nm, and (ii) estimate the average elastic modulus of samples (E), measured in Pa, according to the Derjagin, Muller, Toropov (DMT) model of elastic contact, which is recommended in the case of small tips and stiff samples with small adhesion [43]. All testing was performed in air at ambient conditions (25 °C).

### 2.6. Bacterial Strain and Growth of Biofilms

*Streptococcus mutans* strain UA159 (JM10:pJM1-ldh, luc+, Spc^R^, *luc* under the control of the *ldh* promoter) was utilized as the model organism in the present study. [34,35,42] The selection of antibiotic-resistant colonies was performed on two passages of TH plates (Todd-Hewitt, BD Difco, New Jersey, NJ, USA), supplemented with 0.3% yeast extract (EMD Millipore Sigma, Burlington, MA, USA) and 800 µg/mL of spectinomycin (MP Biomedicals, Santa Ana, CA, USA). The plates were incubated under anaerobic conditions at 37 °C for 48 h. Planktonic cultures of *S. mutans* (JM10) were grown in THY culture medium at 37 °C for 16 h. Cultures having optical density (OD_600_) levels equal to or higher than 0.900 (corresponding to 6.43 e^+12^ CFU/mL) were used as inoculum to grow biofilms. Optimal biofilm growth parameters identified during a previous study from our group [1:50 dilution, 0.65× THY + 1% (*w*/*v*) sucrose, 1000 µL] [6] were then used to grow the biofilms. Aliquots (1.0 mL) of inoculated biofilm growth media were dispensed into the wells of sterile 24-well microtiter plates (Falcon, Corning, NY, USA), containing sterile specimens. Biofilms were grown for 24 h (static cultures, microaerophilic conditions, 37 °C). An additional set of specimens fabricated with OPTB was treated with 2% chlorhexidine gluconate (CHX) for 2 min and served as the control group.

### 2.7. High Throughput Bioluminescence Assay

After the growth period, biofilms were replenished with 1.0 mL of fresh 1^x^ THY + 1% (*w*/*v*) glucose recharge medium and were incubated at 37 °C for 1 h. Replenished biofilms were transferred into the wells of sterile white 24-well plates, containing 1.0 mL of fresh 0.65× THY + 1% (*w*/*v*) sucrose medium. D-Luciferin aqueous solution (100 mM) suspended in 0.1 M citrate buffer (pH 6.0) was added by a computer-controlled system in a Synergy HT Multi-mode microplate reader (Agilent Biotek, Winooski, VT, USA) to the wells containing both the specimens with biofilms and recharge medium in 2:1 ratio (*v*/*v*) of inoculum:D-Luciferin. Luciferase metabolic activity in non-disrupted *S. mutans* biofilms was evaluated at 590 nm in 2 min increments (6 min total) after the addition of D-Luciferin substrate, in terms of relative luminescence units (RLUs). After completion of the assay, the timepoint with the lowest coefficient of variation was selected for results analysis. High throughput bioluminescence procedures reported in the present study were conducted, following a previously published and validated protocol [38].

### 2.8. Long-Term Antibacterial Properties

In the present study, the testing of long-term antibacterial properties was divided into two parts. In part one (simulated shelf-life), unpolymerized adhesive resins identified in Section 2.2. (unaltered [OPTB] and experimental [OPTB + N_TiO_2_) were synthesized and stored (dark conditions, 25 °C) in the original containers provided by the manufacturer (5 mL, black bottles, Kerr Corp., Orange, CA, USA) for the duration of the study (24 months). At four specific time-points (T1 = 0, T2 = 6, T3 = 12 and T4 = 24 months), specimens (*n* = 18/group/time-point; total number of specimens = 90) were fabricated, sterilized, and monomer-extracted, following the procedures and methods described in Section 2.2. Twenty four-hour biofilms were then grown onto the surfaces of specimens, according to protocols described in Section 2.6. and bioluminescence procedures were performed, as described in Section 2.7. In part two (longevity of antibacterial properties), an additional set of specimens (*n* = 18/group/time-point; total number of specimens = 450) were fabricated immediately after the synthesis of experimental adhesive resins. Fabricated specimens were then UV-sterilized, as described in Section 2.2., before being individually stored in 50 mL of sterile PBS (pH 7.4, 37 °C, dark conditions, sealed Falcon tubes) for the duration of the study (12 months). At specific time-points (T1 = 0, T2 = 1, T3 = 3, T4 = 6 and T5 = 12 months) specimens were then subjected to biofilm growth and bioluminescence procedures, as described before.

### 2.9. Statistical Analysis

Values of degree of conversion were statistically analyzed using t-Tests and post hoc Student–Newman–Keuls tests (SAS software, version 9.3; SAS Institute, Cary, NC, USA). Values of biaxial flexure strength were statistically analyzed using general linear models and Student–Newman–Keuls post hoc tests (SAS software, version 9.3; SAS Institute, Cary, NC, USA). Values of elastic modulus and roughness were statistically analyzed using one-way ANOVA and Tukey post hoc tests (JASP, v. 0.15, University of Amsterdam, The Netherlands). RLU values indicating the viability of non-disrupted biofilms of *S. mutans* grown against the surfaces of both unaltered and experimental dental adhesive resins were statistically analyzed using two-factor general linear models (GLM) and post hoc Student–Newman–Keuls tests (SAS software, version 9.3; SAS Institute, Cary, NC, USA). All tests were conducted with a level of significance of 95%.

## 3. Results

**Figure 1:**
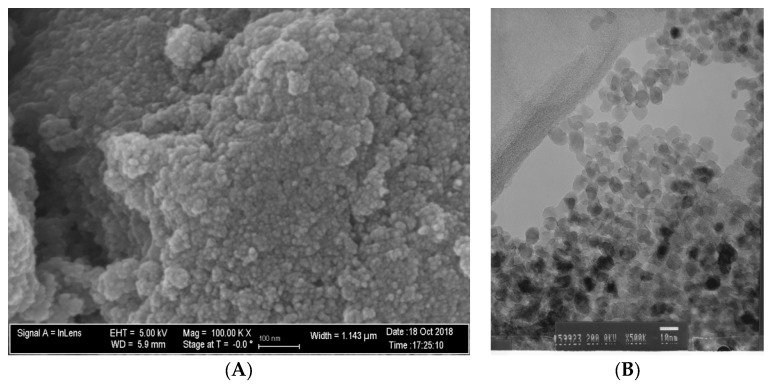
(**A**) SEM (magnification 100 k×) and (**B**) TEM (magnification 500×) micrographs of non-immobilized N_TiO_2_.

Figure 1A,B demonstrate that N_TiO_2_ was produced using solvothermal reactions and, as described in Section 2.1., resulted in nanoparticles that had an approximate spherical shape, had smooth surfaces, and mostly exhibiting some faceting.

**Figure 2:**
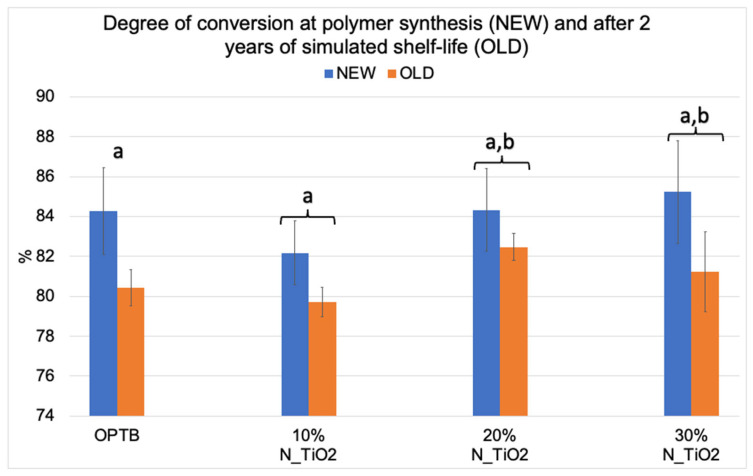
Mean and standard deviation values of degree of conversion (DC) of unaltered (OPTB) and experimental adhesives (OPTB + N_TiO_2_ [10%, 20% or 30%, *v*/*v*]). Connecting braces denote no statistical differences (*p* > 0.05) between NEW and OLD within each group. Letters above bars illustrate the SNK rankings and denote the presence of significant (*p* < 0.05) intergroup differences.

Figure 2 illustrates the mean and standard deviation values of DC for the investigated adhesives (unaltered and experimental). Results reported have clearly indicated that DC values decreased after two years of simulated shelf-life independently of material (unaltered or experimental) or N_TiO_2_ concentration (10%, 20% and 30%) considered. Values of DC varied from 82.17% (NEW, 10% N_TiO_2_) to 85.23% (NEW, 30% N_TiO_2_) and from 79.73% (OLD, 10% N_TiO_2_) to 82.47% (OLD, 20% N_TiO_2_). The smallest reductions in DC values after two years of simulated shelf-life were detected on experimental materials containing 20% N_TiO_2_ (1.86%), which suggests that the incorporation and functionalization of N_TiO_2_ into OPTB does not adversely impact the shelf-life stability of the parental polymer over the course of 24 months. It is well known that restorative materials with low DC values are associated with inadequate properties (physical, mechanical, optical, and biological) and display short service lives. Therefore, these results are critically important, as the experimental materials’ DC has been in congruence with controls within the commercial manufacturer shelf-life specification of two years.

**Figure 3:**
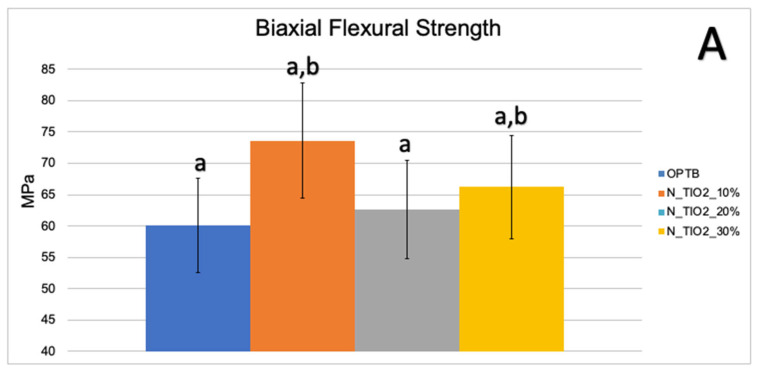

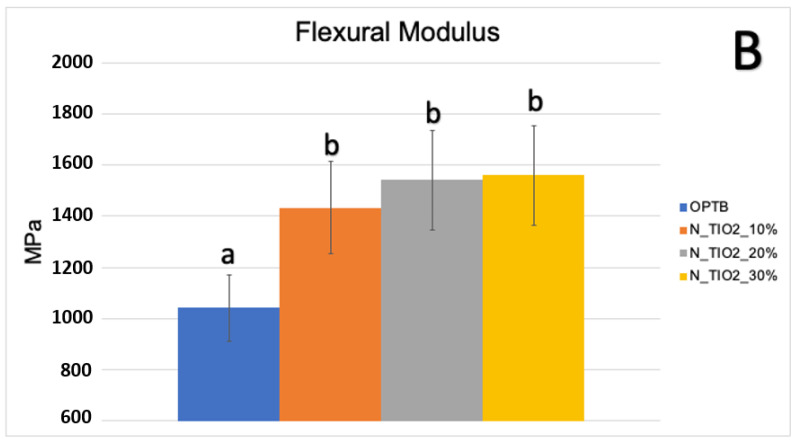
Mean and standard deviation values of (**A**) biaxial flexure strength (BFS) and (**B**) flexural modulus of unaltered (OPTB) and experimental adhesives (OPTB + N_TiO_2_ [10%, 20%, or 30%, *v*/*v*]). Letters above bars illustrate the SNK rankings and denote the presence of significant (*p* < 0.05) intergroup differences.

Figure 3A,B illustrate the results of the biaxial flexure strength test. It can be observed that the incorporation of N_TiO_2_ resulted in experimental materials with superior mechanical properties, as denoted by values of BFS and flexural modulus that were numerically higher and statistically significant (*p* < 0.05; flexural modulus), when compared to those of OPTB. This confirms our hypothesis that experimental materials containing varying concentrations of N_TiO_2_ would display better bulk mechanical properties, when compared to the parental polymer.

**Figure 4:**
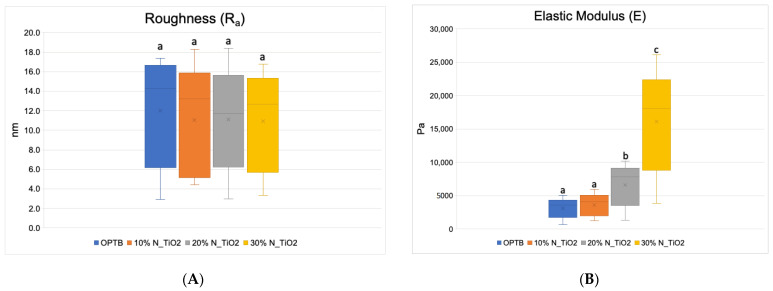

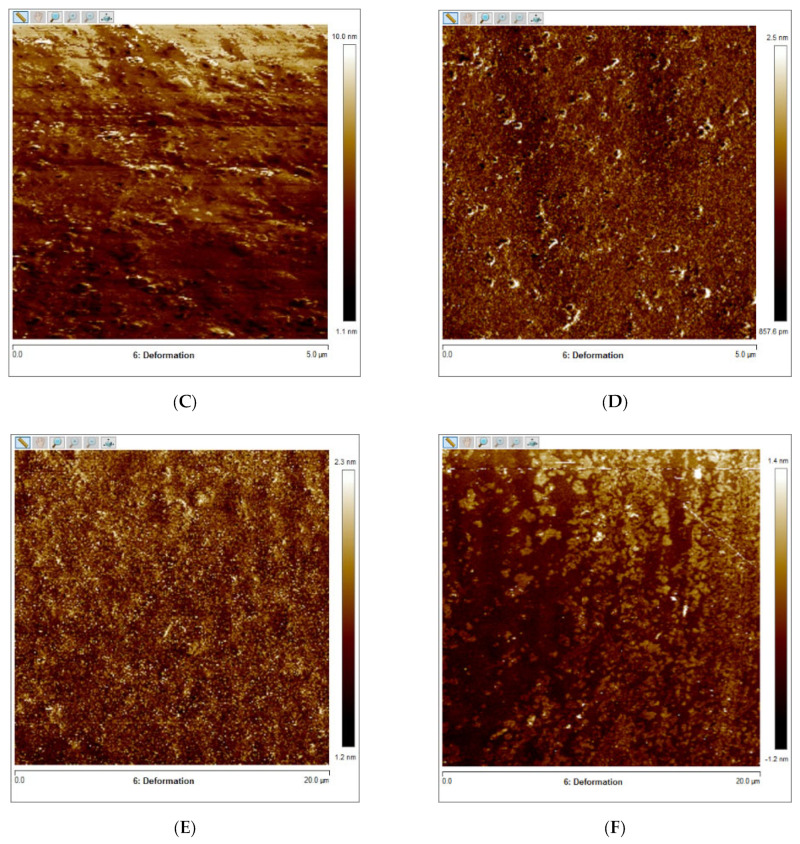
Mean and standard deviation values of (**A**) surface roughness and (**B**) elastic modulus of adhesives containing N_TiO_2_. (**C**–**F**) Raw two-dimensional atomic force images, preceding filters, and post-processing for data analysis, illustrating the mapping of samples deformation during QNM analysis for (**C**) OPTB, (**D**) OPTB+10% N_TiO_2_, (**E**) OPTB+20% N_TiO_2_, and (**F**) OPTB+30% N_TiO_2_. Side bars on individual images illustrate the range of deformation experienced by each material. Letters above bars in images (**A**,**B**) illustrate the SNK rankings and denote the presence of differences that are statistically significant (*p* < 0.05).

Figure 4A–F presents the results of the quantitative nanoscale mechanical characterization of adhesives investigated in terms of surface roughness (4A), elastic modulus (4B), and surface deformation for (C) OPTB, (D) OPTB+10% N_TiO_2_, (E) OPTB+20% N_TiO_2_, and (F) OPTB+30% N_TiO_2_. It is evident that the incorporation of nanoparticles into OPTB resulted in experimental materials capable of producing specimens with surface roughness values that were comparable (*p* = 0.999) to those produced with the parental polymer. The elastic modulus of experimental materials containing 20% and 30% of N_TiO_2_ were demonstrated to be numerically higher and statistically different (*p* = 0.001), when compared to that of the parental polymer, which indicates that experimental materials produced specimens that were much stiffer than those produced with OPTB (control group).

**Figure 5:**
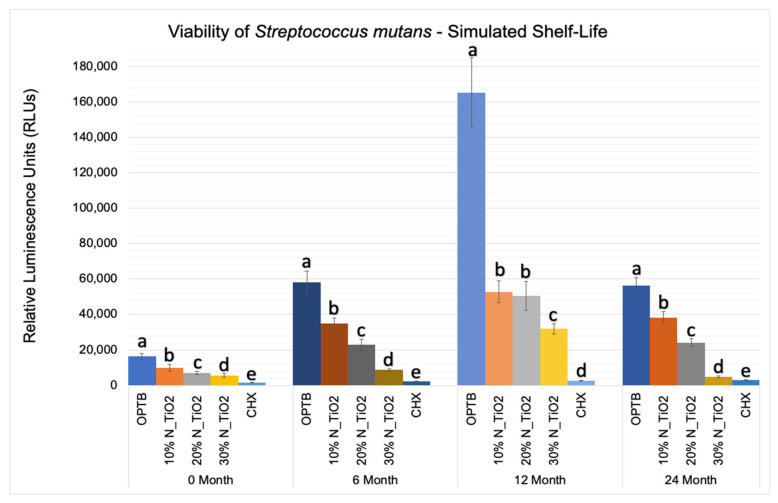
Expression of luciferase activity (*n* = 18/group) was quantified in terms of bioluminescence. Biofilms on specimens that were fabricated from dental adhesives that were aged for 0, 6, 12, and 24 months had sustained lower bioluminescent values when exposed to higher nanoparticle concentrations. CHX is the positive control group. Letters above bars illustrate the SNK rankings and denote mean values that are statistically different (*p* < 0.001) within timepoints of 0, 6, 12, and 24 months.

Figure 5 shows the mean and standard error values of luciferase metabolic activity in terms of relative light units (RLUs) for *S. mutans* biofilms grown on the surfaces of specimens fabricated during the simulated shelf-life portion of the experiment. It can be observed that biofilms grown against the surfaces of OPTB (control group) displayed the highest levels of luciferase metabolic activity amongst all groups investigated and independent of the time-point considered. Twelve-month findings reported for specimens pertaining to the control group (OPTB) should be interpreted with caution because these could have resulted from the intrinsic limitations of the study that include the utilization of a semi-defined biofilm growth medium and the leaching of unreacted hydrophilic monomers [44]. Results reported suggest that experimental materials’ antibacterial properties varied in a concentration-dependent manner, and experimental materials could be rank-ordered in terms of increasing antibacterial properties (10% < 20% < 30%), as denoted by lower RLU values and distinct SNK rankings. The lowest levels of metabolic activity were detected in biofilms topically treated with CHX for 2 min. It can be noted when observing the results from time-points T2, T3, and T4 (6, 12 and 24 months, respectively), that storage time has negatively affected all materials investigated (unaltered and experimental), as denoted by metabolic activities that were progressively higher (in terms of RLU values). It was also possible to observe that experimental materials containing 30% of N_TiO_2_ rendered biofilms with metabolic activities that were comparable to those treated with CHX (at time-points T1 and T4).

**Figure 6:**
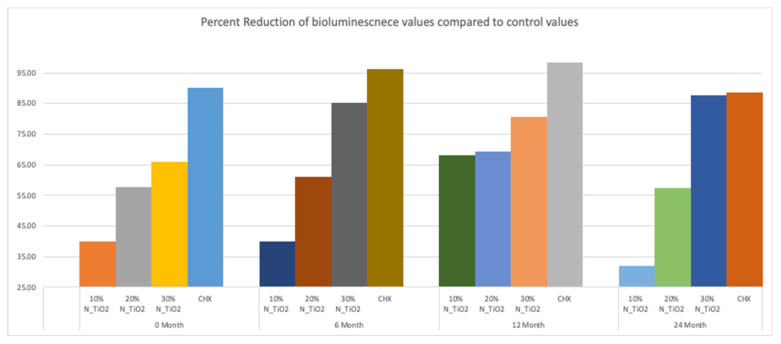
Percent reduction of bioluminescence values of experimental adhesive resins that were fabricated with aged adhesive resins, when compared to unaltered OPTB. Biofilms grown on specimens with higher concentrations of nanoparticles showed greater decrease in bioluminescence. CHX serves as a well-established positive control.

Figure 6 illustrates the percentage reduction in metabolic activity for experimental groups investigated, where it becomes obvious that experimental adhesive resins containing functionalized N_TiO_2_ could sustain long-term antibacterial properties, as denoted by metabolic reductions that ranged from 40% (T1, 10% of N_TiO_2_) to 87% (T4, 30% of N_TiO_2_). The highest reductions in metabolic activity were observed in biofilms treated with CHX (reductions ranging from 88–98%). This was an expected result, because fresh CHX was topically applied onto biofilms at each specific time-point.

**Figure 7:**
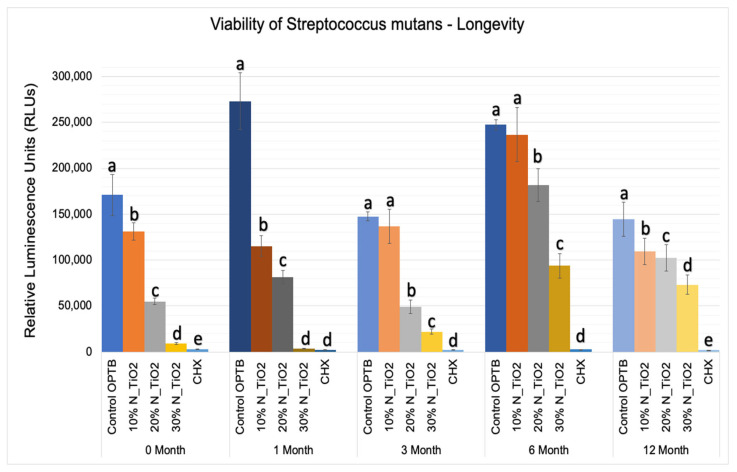
Expression of luciferase activity (*n* = 18/group) was quantified in terms of bioluminescence. Biofilms grown on specimens of dental adhesives that were then aged for 0, 1, 3, 6, and 12 moths in PBS exhibited lower bioluminescent values when exposed to higher nanoparticle concentrations. Letters above bars illuastrate the SNK rankings and denote mean values that are statistically different (*p* < 0.001) within timepoints of 0, 1, 3, 6, and 12 months.

The results of the second part of the experiment (longevity of antibacterial properties) are shown in Figure 7 in terms of mean and standard error values of luciferase metabolic activity (RLUs). In this portion of the study, 24 h *S. mutans* biofilms were grown after specific time-points: T1 = 0, T2 = 1, T3 = 3, T4 = 6, and T5 = 12 months; on the surfaces of specimens (in PBS, 37 °C, duration of study); fabricated with unaltered (OPTB) or experimental adhesive resins containing varying concentrations of N_TiO_2_ (10%, 20% or 30%, *v*/*v*%).

Results reported in Figure 7 agree with those from Figure 5 and Figure 6 and suggest that experimental materials’ antibacterial properties varied in a concentration-dependent manner, where materials containing higher N_TiO_2_ concentrations displayed antibacterial properties (10% < 20% < 30%) that were stronger, as denoted by RLU values that were consistently smaller and statistically different (*p* < 0.001) than those from control group specimens (OPTB). The antibacterial properties of experimental adhesive resins containing 30% of N_TiO_2_ were comparable to those from CHX (topical treatment, 2 min) at time-points T1 and T2 (0 and 1 month) but were less pronounced in time-points T3, T4, and T5 (3, 6, and 12 months, respectively).

**Figure 8:**
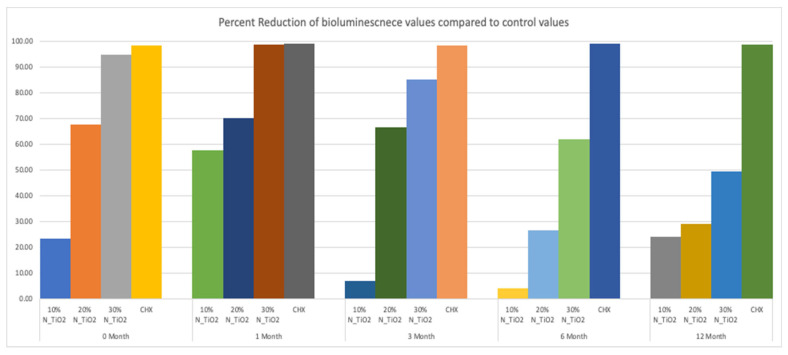
Percent reduction of bioluminescence values of experimental adhesive resins that were fabricated and aged in PBS, when compared to unaltered OPTB. Biofilms grown on fabricated specimens that exhibited higher concentrations of nanoparticles showed greater decrease in bioluminescence, however the percent reduction decreases over time. CHX is to serve as a well-established positive control.

This less-than-ideal behavior indicates that long-term storage in PBS adversely impacts the properties of the materials investigated. However, when comparing these results to those from the control group, it is obvious that experimental materials proposed were able to sustain relevant antibacterial properties against non-disrupted *S. mutans* biofilms. Figure 8 helps to further illustrate that point by displaying the results of metabolic reduction (in %) among experimental groups, when compared to the control (OPTB). The group treated with CHX displayed the most consistent and effective antibacterial behavior amongst all groups investigated and displayed viability reductions that ranged between 98 and 99%. Biofilms grown against the surfaces of specimens fabricated with experimental adhesive resins displayed metabolic reductions that ranged from 4.36% (T1, 10% of N_TiO_2_) to 98.63% (T2, 30% of N_TiO_2_). In combination, reported results indicate that experimental materials were indeed capable of sustaining relevant long-term (up to 24 months) antibacterial properties against non-disrupted *S. mutans* biofilms without the need for visible light irradiation.

## 4. Discussion

Several approaches have been tested to impart long-term antibacterial properties to dental adhesive resins. These include the utilization of fluorinated graphene [45], eugenyl methacrylate (EgMA) [46], chlorhexidine (0.2% diacetate or 2% digluconate) [47], tt-farnesol [48], sodium hypochlorite (6%) [49], benzalkonium chloride [50], and epigallocatechin-3-gallate [51], among others. However, even though experimental materials were able to initially decrease the viability of oral microorganisms, reports have indicated that newly developed materials display a low degree of polymerization, reduced mechanical properties, and leaching of uncured monomers [52] and were not able to display significant long-term antibacterial properties, or were capable of extending the service lives of polymer-based adhesive restorations. The photocatalysis of metaloxide nanoparticles has been considered as an alternative approach [53,54] because of their proven effectivity against microorganisms relevant to public health [34]. However, UV-irradiation requirements have restricted its widespread use in dental applications and resulted in materials displaying rough surfaces and degraded polymer matrixes. [25,55] Subsequent studies investigated the antibacterial efficacy of heterogeneous photocatalysis and doped nanoparticles against *S. mutans* biofilms [34]. According to results reported, experimental adhesives containing N_TiO_2_ (50%, 67% and 80%) displayed strong antibacterial and biomimetic properties when irradiated with visible light (410 ± 10 nm, 310.07 J/cm^2^). Specimens fabricated with experimental adhesives reported were shown (SEM/EDX data) to have smooth surfaces and polymer matrixes that were not degraded [34].

Follow-up studies investigated if relevant antibacterial properties could be achieved in the absence of visible light and to determine the impact of N_TiO_2_ incorporation on the sorption, solubility, and biocompatibility of experimental adhesives [20]. Results indicated that experimental adhesives were less soluble, absorbed less water, were more biocompatible and more antibacterial, when compared to the parental polymer (OPTB). When comparing experimental materials’ antibacterial properties to those of Clearfil SE Protect (Kuraray, Co.), which is a commercially available, MDPB-containing, and fluoride-releasing dental adhesive resin, experimental adhesives containing N_TiO_2_ displayed comparable (*p* > 0.05) efficacies against non-disrupted *S. mutans* biofilms (either 24 or 48 h) in dark conditions, [38] thereby demonstrating that the nanotechnology proposed could impart relevant toxicity against *S. mutans* without visible light irradiation. Despite these promising results, previous studies failed to investigate other relevant properties of experimental adhesives containing N_TiO_2_ (10%, 20%, and 30%), including the degree of conversion at the time of polymer synthesis and after two years of simulated shelf-life, the biaxial flexure strength, flexural modulus, surface roughness, elastic modulus, and long-term antibacterial properties (12 and 24 months).

The rationale for fabricating N_TiO_2_ using solvothermal reactions under supercritical conditions of temperature and pressure precipitates from the fact that this synthetic route has been previously shown [56] to be highly reproducible and to yield pure and crystalline nanoparticles (6–15 nm, anatase phase) with high levels of nitrogen doping (N/Ti molar ratio = 3.4%), when compared to traditional calcination strategies (N/Ti molar ratio = 1.3%). Nanoparticles fabricated by this process are electron deficient, display smooth surfaces, have high specific surface area, well-defined pore architecture, can generate substantial amounts of ROS [56], and absorb twice as much visible light, when compared to their calcinated and undoped counterparts [34]. The synthesis of experimental adhesives was conducted using a previously published protocol because such a route allows for the functionalization of non-agglomerated N_TiO_2_ and results in experimental materials with proven antibacterial properties [56]. Figure 2 illustrates that the functionalization of N_TiO_2_ into OPTB, which is a self-etch and 5th generation dental adhesive resin, resulted in experimental materials displaying DC values that were either comparable or higher than those from the parental polymer. In addition, the reported results have indicated that experimental adhesives containing 20% of N_TiO_2_ displayed the smallest decrease in DC values after two years of simulated shelf-life, thereby indicating that the nanotechnology proposed does not adversely impact the stability of the parental polymer and has the ability to maintain DC values at levels that are considered clinically relevant.

Mohammed and Riad, [57] while investigating the effects of silver nanoparticles (6.25, 12.5, 25, 50 and 100 μg/mL) on the antibacterial properties and degree of conversion of a self-etch adhesive resin (Universal Bond, 3M ESPE, St. Paul, USA), have demonstrated that the incorporation of commercially available nanoparticles (in ethanol suspension or powder) resulted in experimental materials with DC values (26.14% ± 4.47% [in suspension] and 47.72% ± 4.47% [powder]) that were numerically lower and statistically different (*p* < 0.001, in suspension) than the DC values from the control group (50.31% ± 4.04%, no nanoparticles). These findings have highlighted the importance of the present study’ results because the authors [57] have clearly demonstrated that simple incorporation of metaloxide nanoparticles into existing polymer formulations does not necessarily lead to the development of materials with improved properties, such as the ones reported herein. The biaxial flexure strength and flexural modulus data presented in Figure 3A,B indicate that the incorporation of N_TiO_2_ into OPTB resulted in experimental materials displaying improved mechanical properties. The findings of the present study have been corroborated by Giannini et al. [58] while investigating the effects of filler particles on the mechanical properties of commercially available (Adper Single Bond [3M ESPE] and Prime and Bond NT [Dentsply]) dental adhesive resins. According to results reported [58], materials containing filler particles in their compositions displayed values of biaxial flexural strength and modulus that were higher to those from unfilled adhesive resins.

The results from the nanoscale mechanical characterization (Figure 4A–F) of adhesives investigated have indicated that the functionalization of N_TiO_2_ into OPTB resulted in experimental materials with surface properties that were comparable to those of the parental polymer. These results are fundamentally important from the mechanical and biological standpoints because it is well known that increased surface roughness results in larger surface areas, higher aggregation, and growth of oral biofilms, and to premature mechanical failures. Results reported have also demonstrated that experimental materials containing 20% and 30% of N_TiO_2_ had elastic moduli that were significantly (*p* < 0.001) higher than that of OPTB, which indicates that experimental materials could potentially withstand the harsh conditions of the oral cavity. The results of the present study have been corroborated by the findings recently reported by Azmy et al. [59] who demonstrated that the incorporation of nanoparticles (ZrO_2_, TiO_2_, and SiO_2_) into dental polymers resulted in experimental materials displaying superior wear resistance and flexural strength, thereby further supporting the experimental design and the nanotechnology proposed here.

Figure 5 illustrated that experimental adhesives investigated in the present study decreased, in a concentration-dependent manner, the viability of non-disrupted *S. mutans* biofilms (24 h) grown against the surfaces of specimens fabricated after specific periods of simulated shelf-life (T1 = 0, T2 = 6, T3 = 12 and T4 = 24 month). Results in Figure 6 illustrates that the attained reductions in metabolic activity (30% of N_TiO_2_) were similar to those observed from biofilms topically treated with CHX (at T1 and T4).

The second part of the experiment was designed to demonstrate if specimens fabricated (immediately after the process described in Section 2.2) and stored in PBS (37 °C, duration of study) would be able to sustain strong and long-term (longevity) antibacterial properties. It is possible to observe in Figure 7 that experimental adhesives decreased, in a concentration-dependent manner, the metabolic activity of non-disrupted *S. mutans* biofilms independently of the time-point considered (T1 = 0, T2 = 1, T3 = 3, T4 = 6, and T5 = 12 months). Figure 8 shows that adhesives with 30% of N_TiO_2_ displayed long-term antibacterial properties that were comparable to CHX (at time-points T1 and T2). Even though PBS storage was observed to decrease the antibacterial efficacy of all materials investigated, metabolic viability reductions (at T3, T4, and T5) attained with 30% of N_TiO_2_ ranged from 49.30% (at T5) to 85.11% (at T3), which is still considered significant. Careful consideration must be exercised by the reader when comparing the antibacterial efficacies attained with either CHX (reductions ranged from 88% to 98%) or experimental adhesives investigated (4.36% [T1, 10% of N_TiO_2_] to 98.63% [T2, 30% of N_TiO_2_]) because fresh CHX (2%) was topically applied (for 2 min) onto biofilms immediately before bioluminescence procedures.

Taken together, the results of the present study have shown for the first time that synthetic strategies adopted (for nanoparticles and experimental adhesives) translated into experimental adhesives displaying promising non-leaching and long-term antibacterial properties (up to 24 months) without the need for visible light irradiation. Furthermore, specimens fabricated using experimental materials reported in the present study displayed surface characteristics that were similar to those fabricated with the parental polymer (OPTB) and did not exhibit any evidence of phase separation between the polymer matrix and nanoparticles during the periods of simulated shelf-life investigated. The absence of phase separation is a strong indication of the successful incorporation and functionalization of nanoparticles into commercially available dental polymers and may lead to materials displaying superior biological, mechanical, and physical properties [39].

The results of the present study have been corroborated by Melo et al. [60] while investigating the long-term antibacterial properties of experimental dental adhesive resins containing silver nanoparticles (0.05%) and cationic quaternary ammonium monomers (0–40%). According to the reported results [60], specimens (n = 6/group; diameter = 9.0 mm) were able to significantly (*p* < 0.05) decrease CFU/mL counts of *S. mutans*, total *Streptococci,* and total microorganisms in a dental plaque biofilm model after 24 h and after 6 months. Zhang et al. [61] investigated the long-term antibacterial properties of a novel bonding agent containing dimethylaminohexadecyl methacrylate (DMAHDM) after aging in water (37 °C; 1, 30, 90 and 180 days) and reported [61] that experimental materials were able to sustain significant (*p* < 0.05) antibacterial properties after 6 months. Machado et al. [62] recently investigated the long-term efficacy (up to 6 months, water storage, 37 °C) of experimental adhesive resins (66.66% Bis-GMA and 33.33% HEMA) containing triclosan-loaded chitosan particles (TLP) against *S. mutans* biofilms, and reported that experimental materials containing higher concentrations of TLP displayed significantly (*p* < 0.05) lower CFU/mL values (for planktonic cultures and biofilms), when compared to the parental polymer (without TLP) [62]. These results further corroborate the present study’s rationale for the utilization of functionalized and non-agglomerated nanoparticles displaying non-leaching and long-term antibacterial properties (up to 24 months).

Future studies from our group will investigate (i) the long-term antibacterial and biomimetic properties of experimental adhesive resins containing third-generation co-doped metaloxide nanoparticles, (ii) the mechanisms of action by which immobilized nanoparticles downregulate the viability of cells and (iii) what genes and metabolic pathways are adversely affected by experimental materials containing single- or co-doped nanoparticle.

## 5. Conclusions

The present study has reported the synthesis of nitrogen-doped titanium dioxide nanoparticles and their successful incorporation into a commercially available dental adhesive resin. Experimental adhesives were demonstrated to display higher values of degree of conversion after polymer synthesis and after two years of simulated-shelf life and to display better mechanical properties. The antibacterial efficacies of experimental materials reported were comparable to CHX at specific time-points and sustained strong antibacterial properties during extended periods of simulated shelf-life (24 months) and aging in PBS (12 months). The results of the present study suggest that experimental materials reported may extend the service lives of polymer-based bonded restorations by decreasing the viability of pathogenic oral microorganisms, typically associated with the occurrence of secondary caries.

## Data Availability

Datasets generated and analyzed in the present study are available from the corresponding author on reasonable request.
